# Biodiversity of Fungi in Freshwater Ecosystems of Italy

**DOI:** 10.3390/jof9100993

**Published:** 2023-10-07

**Authors:** Giulia Mirabile, Valeria Ferraro, Francesco Paolo Mancuso, Lorenzo Pecoraro, Fortunato Cirlincione

**Affiliations:** 1 Department of Agricultural, Food and Forest Sciences, University of Palermo, Viale delle Scienze, Bldg. 5, 90128 Palermo, Italy; giulia.mirabile@unipa.it (G.M.); fortunato.cirlincione@unipa.it (F.C.); 2 NBFC, National Biodiversity Future Center, Piazza Marina 61 (c/o Palazzo Steri), 90133 Palermo, Italy; 3 Department of Pharmacy-Pharmaceutical Sciences, University of Bari “Aldo Moro”, University Campus “Ernesto Quagliariello”, Via E. Orabona 4, 70125 Bari, Italy; 4 Department of Earth and Sea Sciences, University of Palermo, Viale delle Scienze, Bldg. 16, 90128 Palermo, Italy; 5 School of Pharmaceutical Science and Technology, Tianjin University, Tianjin 300072, China; lorenzo.pecoraro@tju.edu.cn

**Keywords:** freshwater fungi, fungal biodiversity, basidiomycota, ascomycota, freshwater ecosystems

## Abstract

Fungal biodiversity is still mostly unknown and their presence in particular ecosystems such as freshwater habitats is often underestimated. The ecological role that these fungi play in freshwater environments mainly concerns their activity as decomposers of litter and plant material. At present, it is estimated that 3870 species belong to the ecological group of freshwater fungi (13 phyla and 45 classes). In this survey, we provide an overview of the Italian freshwater fungal diversity on the basis of the field and literature data. In the literature, data on freshwater fungi are fragmentary and not updated, focusing mainly on northern Italy where the most important lakes and rivers are present, while data from central and southern Italy (including Sicily and Sardinia) are almost completely ineffective. In particular, Ascomycota are reported in only 14 publications, most of which concern the freshwater environments of Lombardia, Piemonte, and Veneto. Only one publication explores the biodiversity of freshwater Basidiomycota in the wetlands of the Cansiglio forest (Veneto). The field observation allowed for us to identify 38 species of Basidiomycota growing in riparian forest of Italy. However, the number of fungi in freshwater habitats of Italy is strongly underestimated and many species are still completely unknown.

## 1. Introduction

Freshwater fungi are an important and ubiquitous group of taxa that includes any species which spend their whole life cycle, or part of it, in freshwater habitats (ponds, pools, lakes, peat swaps, wetlands, rivers, streams, artificial reservoirs, etc.) or which colonize the submerged part of plants in freshwater environments [1,2,3]. Currently, only about 3000–4000 species of fungi have been classified as aquatic fungi. Considering that estimations of global fungal diversity range from 0.5 to 10 million species, our knowledge of this group of fungi is still very limited. Indeed, some taxonomic groups are almost entirely uninvestigated, and many aquatic habitats are still unexplored [3,4,5]. Freshwater fungi are separated into different groups according to their morphology and ecology: freshwater ascomycetes, freshwater hyphomycetes (“Ingoldian fungi”, aero-aquatic hyphomycetes, terrestrial-aquatic hyphomycetes, submerged-aquatic hyphomycetes), freshwater basidiomycetes, coelomycetes, zygomycetes, microsporidia, and zoosporic fungi [2,6,7]. The evolution of molecular approaches used for fungal identification has led to a rapid change in their classification, with the introduction of new genera, families, orders, and classes of freshwater fungi. At present, they are known belong to 13 fungal phyla: Aphelidiomycota, Ascomycota, Basidiomycota, Blastocladiomycota, Chytridiomycota, Monoblepharomycota, Mortierellomycota, Rozellomycota, Mucoromycota, Entomophtoromycota, Olpidiomycota, Zoopagomycota, and Sanchytriomycota [3,8,9,10]. Most taxa belong to Ascomycota (2968 species) and Chytridiomycota (333 species), while Basidiomycota (218 species) are rarely isolated from aquatic environments [9,11]. Data collection on freshwater fungi mostly derive from temperate areas of Asia, Australia, North America, and Europe, but they can also have a cosmopolitan distribution, or they can grow in tropical or cold-water habitats [7,12].

Many of the fungal species belonging to the three major groups of freshwater fungi (Ascomycota, Chytridiomycota, and Basidiomycota) are best known for being important plant pathogens, parasites or symbionts. Their presence in aquatic environments, however, is related to their key role as decomposers of dead plant and animal biomasses [13,14,15]. Most freshwater fungi can decompose a wide range of substances, transferring nutrients to higher trophic levels of the food web. In benthic lake environments, leaf-colonizing fungi improve the palatability of litter for macrozoobenthos grazers [16]. Overall, most aquatic hyphomycetes play an important role in the decomposition of plant materials (leaves or herbaceous debris), degrading cellulose, pectins, and hemicellulose, while ascomycetes and basidiomycetes can break down wood (lignicolous or wood decay freshwater fungi) [7,17,18,19,20]. Hyphomycetes, in particular, upgrade the nutritional value of leaf litter by lowering the carbon–nitrogen–phosphorus ratio [21]. The decomposition of plant litter in freshwater habitats depends on environmental (pH, temperature, oxygen presence/absence) and hydrologic conditions (plant litter constantly exposed to air, or constantly submerged, partially submerged, etc.) [22,23]. Smaller substrates containing chitin, cellulose or keratin, such as seeds, algae, and zooplankton carcasses, are mainly decomposed by the saprobes zoosporic fungi Chytridiomycetes and Oomycetes, rather than the other fungal groups.

Often, zoosporic fungi can act as parasites. In case of parasitism, zoosporic fungi can cause severe damage to aquatic organisms such as fishes, frogs and crustacea. Moreover, some oomycetes (e.g., *Pythium* sp.) can severely affect aquatic plants [24,25,26,27].

Freshwater fungi are directly in involved both carbon and nitrogen cycles. During plant litter decomposition, as a consequence of the respiratory process, freshwater fungi convert plant carbon in fungal biomass and CO_2_, contributing to the carbon cycle. Regarding nitrogen, aquatic fungi can utilize nitrogen from water and organic substrates (especially submerged wood), increasing their biomass and the sporulation process [28].

As symbionts, freshwater fungi can form a mycorrhizal relationship with the roots of aquatic plants, trees, and macrophytes. The first report of arbuscular mycorrhizal fungi in aquatic plants was provided by Søndergaard and Laegaard [29] and, among macrophytes, isoetids are those with the highest level of mycorrhizal colonization. Thanks to this relationship, aquatic plants provide nutrients for the mycorrhizal fungus, and this makes nutrients available to the plants [30].

Considering their fundamental role in the ecosystem, the protection and conservation of freshwater fungal biodiversity is one of the objectives of the National Biodiversity Future Center (NBFC) financed by the National Recovery and Resilience Plan (NRRP). One of the goals of NBFC (Spoke 3, Biodiversity) is to improve the basic knowledge of freshwater fungal biodiversity in the Mediterranean areas. This study aims to analyze the knowledge of freshwater fungal biodiversity (with the exclusion of yeasts, Chromista and lichens) in Italy.

## 2. Materials and Methods

### 2.1. Literature Data

The Italian territory is divided into continental, peninsular, and insular sectors. The continental sector is delimited by the Italian Alps and the upper part of the Appennines; the peninsular one extends from the Tuscan–Romagna Appennines to Calabria and is delimited by the Ligurian Sea, Thyrrenian Sea, Ionian Sea, and Adriatic Sea, while the insular part comprises the two largest islands, Sicily and Sardinia, and about 800 islets. In the Mediterranean Basin, Italy is the richest territory in terms of freshwater habitats, with 69 natural lakes and 183 artificial basins, respectively, and ca. 230 rivers and streams [31]. The importance of protecting the biodiversity of these ecosystems is increasing, but while most of the research considers plant communities, the knowledge regarding freshwater Italian fungi is extremely restricted and fragmented. A piece of online research was carried out to find any published data about freshwater fungi reported in Italy from the XIX century to present. The research was carried out using both English and Italian terms, using online databases such as Scopus, Web of Sciences, and Google Scholar. The considered data include both macro- and micromycetes reported in freshwater habitats (lakes, rivers, wetlands, ponds, lagoons, streams). As mentioned before, lichens, Chromista and yeasts were not included in the data research.

### 2.2. Field Data

Field data collection concerned only macromycetes in order to fill an obvious gap in the studies conducted to date. Weekly and fortnightly observations were carried out during the autumn/winter/spring seasons, and monthly observations were carried out during summer in different freshwater ecosystems of Italian regions. For each observed species, fresh basidiomata were collected and identified by morphological analysis using analytical keys. Macroscopic features such as lamellae, pores, stipe, flesh, pileus, spore prints, type of occurrence (cespitose, solitary, clustered, grouped), etc., were observed by naked eye and by a stereoscopic microscope when needed, while microscopic features such as spores, basidia, hyphae, pileipellis, cystidia, etc., were observed using an optical microscope with the help of chemical reagents (KOH, ammoniated Congo Red, Melzer’s reagent, Cotton blue-lactic acid), distilled water and immersion oil when necessary [32]. Finally, basidiomata were dried in a universal dryer with 475 Watt stainless steel and stored in the Herbarium SAF of Department of Agricultural, Food and Forest Sciences (SAAF) of the University of Palermo. For fungal and plant nomenclature, the Index Fungorum database and Euro + Med PlantBase databases were consulted.

## 3. Freshwater Fungi

### 3.1. Freshwater Ascomycota

Thirteen classes belong to this group of fungi: Arthoniomycetes, Candelariomycetes, Coniocybomycetes, Dothideomycetes, Eurotiomycetes, Laboulbeniomycetes, Lecanoromycetes, Leotiomycetes, Lichinomycetes, Orbiliomycetes, Pezizomycetes, Saccharomycetes, and Sordariomycetes, and ca. 3000 species. This group includes genera that live exclusively in freshwater habitats, fungi that live both in freshwater and terrestrial habitats, fungal species that live in freshwater and marine habitats, and species that are found in freshwater, terrestrial, and marine habitats [33]. Among Ascomycota, Sordariomycetes, and Dothideomycetes represent the largest classes. Sordariomycetes comprise 823 freshwater species distributed in 298 genera. Among the 40 orders of Sordariomycetes, Chaetosphaeriales is the largest, with 149 species recorded from freshwater habitats. Microscopically, they are characterized by inoperculate unitunicate asci and perithecial ascomata [10,34,35]. They play an important role in freshwater habitats, decomposing lignocellulose substances in submerged woody litter, and some of them are also able to produce bioactive compounds [19].

Dothideomycetes comprises 18 orders, 75 families, and 229 genera [10]. Recent studies have reported 677 species of Dothideomycetes isolated from freshwater habitats. Among them, 391 species belong to Pleosporales, the largest freshwater order of Dothideomycetes. They are characterized by bitunicate asci contained in ascostromatic ascomata. Often, asci present fissitunicate dehiscence, which is typical of this class [6,9,10,36]. Dothideomycetes are mostly saprobic in freshwater habitats, decomposing submerged leaves and woody debris in lentic or lotic habitats [37].

### 3.2. Freshwater Chytridiomycota

A total of 97 genera and 333 species belong to the phylum Chytridiomycota which also includes nine classes: Chytridiomycetes, Cladochytriomycetes, Lobulomycetes, Mesochytriomycetes, Polychytriomycetes, Rhizophlyctidomycetes, Rhizophydiomycetes, Spizellomycetes, and Synchytriomycetes. The largest class is represented by Chytridiomycetes, with 181 species [38]. They are commonly named chytrids and are characterized by the presence of an uniflagellate zoospore in the back part of the asexual propagule. Many chytrids need to produce zoospores to keep growing, and others can survive in the cold and periods of desiccation by forming resting sporangia. Chytrids can degrade chitin, cellulose, lignin, and keratin and can act like saprotrophs or parasites on algae, fungi, plants, insects, mosses, and invertebrates [11].

### 3.3. Freshwater Basidiomycota

Compared to Ascomycota, a small number of Basidiomycota have been isolated from freshwater habitats. The group comprises 11 classes (Agaricomycetes, Agaricostilbomycetes, Bartheletiomycetes, Classiculomycetes, Cystobasidiomycetes, Exobasidiomycetes, Microbotryomycetes, Moniliellomycetes, Tremellomycetes, and Ustilaginomycetes), 100 genera, and 218 species. Tremellomycetes is the class with the highest number of species (81) isolated from freshwater habitats [10]. Freshwater Basidiomycota include filamentous fungi, endophytes, and saprobic yeasts. Fungi belonging to these groups are characterized by the presence of binucleate cells or clamp-connections [39]. Most of them utilize simple carbohydrates, while filamentous species are able to decompose cellulose, hemicellulose, and lignin. For these reasons, they can be found on a great variety of substrates, such as the culms of freshwater plants (e.g., *Equisetum* sp.), submerged wood, decaying leaves, water, and foams [39,40].

### 3.4. Freshwater Hyphomycetes

Freshwater Hyphomycetes are also known as Ingoldian Hyphomycetes, in honor of Prof. Cecil Terence Ingold, who, in 1942, discovered a typical habitat for these fungi, growing on submerged decaying leaves belonging to broad-leaved trees in well-aerated waters [41]. This is a dominant group (95 genera and 330 species) in freshwater habitats formed by an asexual morph of Ascomycota and Basidiomycota [42]. They are characterized by the production of modified hyaline conidia with unique shapes: tetra-radiate, filiform, multiradiate, sigmoid, branced, and solecoid [2,43,44]. They can be found in fast-flowing streams, well-aerated lakes, and humid environments growing on submerged decaying plant material [41,45]. Most of them belong to Ascomycota (Leotiomycetes, Dothideomycetes, Orbiliomycetes, Sordariomycetes), while few taxa are reported in Basidiomycota.

## 4. Results

### 4.1. Literature Data

In Table 1, the freshwater Ascomycota isolated from different substrates and freshwater habitats along the Italian territory are listed. The list counts 126 species of freshwater Ascomycota belonging to Chytridiomycetes, Dothideomycetes, Eurotiomycetes, Leotiomycetes (the most numerous), Laboulbeniomycetes, Pezizomycetes, and Sordariomycetes classes. The first reports in Italy were presented by Saccardo [46] in the Sylloge Fungorum. He described a species belonging to the class Chytridiomycetes on an unidentified submerged decaying wood sampled in Canal Grande (Veneto) and four species belonging to the class Laboulbeniomycetes on the bodies and legs of aquatic insects in De Tarzo Lake and in some ditches in Conegliano (Veneto). In 1959, Ciferri listed 11 species of aquatic Hyphomycetes isolated from Ticino and Po basins and in canals of irrigation located in Pavia (Lombardia). No other data were presented until 1983, when Del Frate and Caretta published a survey carried out of a permanent mountain stream in Valsesia (Piemonte) from which they isolated 20 species of freshwater Hyphomycetes belonging to Dothideomycetes (*Anguillospora crassa* Ingold, *A. longissima* (Sacc. and P. Syd.) Ingold, *Clavariopsis aquatica* De Wild, *Tripospermum myrti* (Lind) S. Hughes), Leotiomycetes (*Alatospora acuminata* Ingold, *Articulospora inflata* Ingold, *A. tetracladia* Ingold, *Flagellospora curvula* Ingold, *Lemonniera centrosphaera* Marvanová, *L. terrestris* Tubaki, *Tetrachaetum elegans* Ingold, *Tricladium attenuatum* S.H. Iqbal, *T. splendens* Ingold, *Varicosporium elodeae* W. Kegel), and other species included in *Incertae sedis*. Abdullah and coworkers [47,48], reported two new species of aero-aquatic fungi belonging to Leotiomycetes (*Pseudaegerita ossiformis* Abdullah, Gené and Guarro and *Spirosphaera lignicola* Abdullah, Gené and Guarro) isolated from unidentified submerged decaying twigs in a stream located in Monticiano (Tuscany). Rodino and coworkers [49] reported 32 identified freshwater fungal taxa from a canal of Ticino Park (Lombardia), with 15 species described for the first time in Italy (*Actinosporella megalospora* (Ingold) Descals, Marvanová and J. Webster, *Articulospora grandis* Greath, *Camposporium pellucidum* (Grove) S. Hughes, *Campylospora parvula* Kuzuha, Heliscella stellata (Ingold and V.J. Cox) Marvanová, *Lemonniera filiformis* R.H. Petersen ex Dyko, *Mycocentrospora aquatica* S.H. Iqbal, *Polycladium equiseti* Ingold, *Pyramidospora casuarinae* Sv. Nilsson, *Speiropsis* sp., *Triscelophorus monosporus* Ingold, *Tumularia aquatica* (Ingold) Descals and Marvanová, *Varicosporium delicatum* S.H. Iqbal). Later, Angelini and coworkers [50], Duarte and coworkers [51], and Davolos and coworkers [52] published the first surveys on freshwater Ascomycota in lakes and rivers from central Italy (Trasimeno Lake, Umbria; Bracciano Lake and Sacco River, Lazio). Finally, other studies about freshwater Ascomycota were published by Bizio and Borsato [53], and Gruppuso and coworkers [54], carried out in Cansiglio forest (Veneto), and Alpine streams of Piemonte, respectively.

Very little data are available on freshwater Basidiomycota from Italy. The only survey found in the literature was carried out by Bizio and Borsato [53]. They reported the presence of 145 Basidiomycetes associated with litter and portions of *P. abies*, *A. sylvestris*, *F. ulmaria*, *A. incana*, *F. sylvatica*, *S. caprea* and other plant communities grown along freshwater habitats of the Cansiglio forest (Veneto).

### 4.2. Field Observations

Macroscopic and microscopic analyses allowed for the identification of 38 species of Basidiomycota growing along the banks and vegetation of Italian lakes and rivers (Table 2).

**Figure 1:**
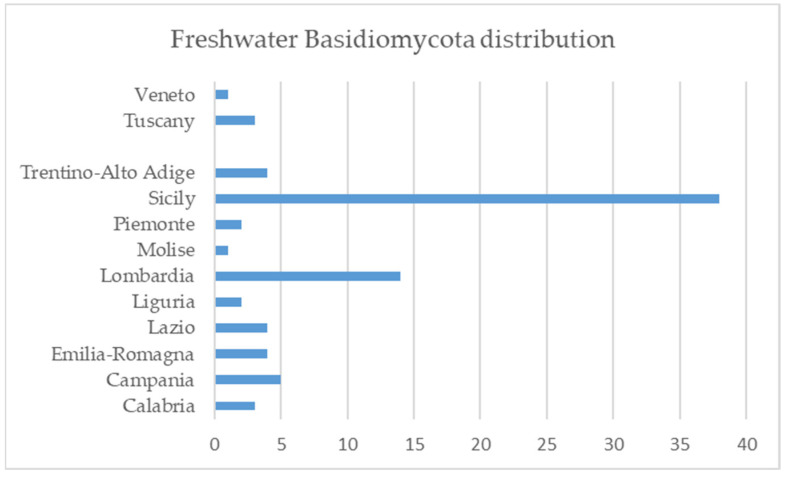
Number of macromycetes per Italian region.

All the reported species were observed in riparian forests along the most important Sicilian rivers, while 14 of them were observed in Lombardia (Figure 1) in the forests along the banks of Lugano, Varese and Bernigolo lakes, and shoreline of Oglio and Ticino rivers.

**Figure 2:**
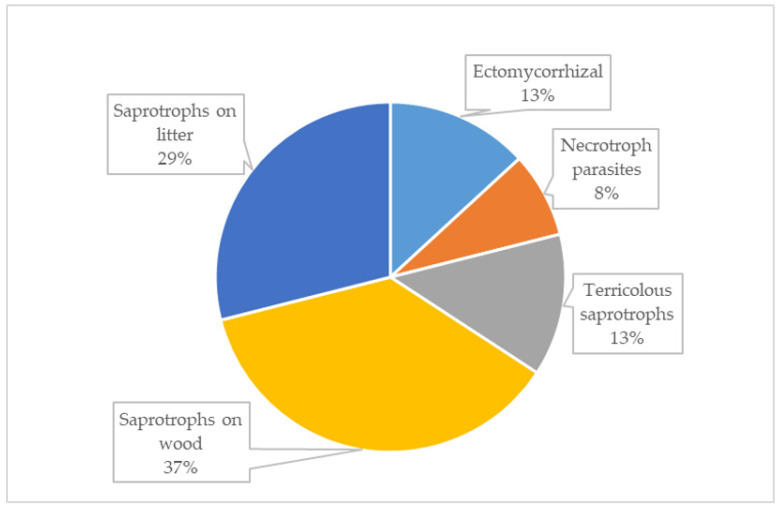
Ecological categories: percentage of mycorrhizal, necrotroph parasites and saprotrophs species of macromycetes growing in freshwater habitats of Italy.

As reported in Figure 2, the observed taxa belong to five ecological categories associated with typical riverbed vegetation, such as *Tamarix africana*, *Populus alba*, and *Salix alba*. Saprotrophs on wood (14 species) are the most numerous ecological category, followed by saprotrophs on litter (11 species), ectomycorrhizal and terricolous saprotrophs (5 species, respectively), and necrotroph parasites (3 species).

## 5. Discussion

As in terrestrial environments, fungi also have a fundamental ecological role in freshwater environments. In recent years, studies about their distribution and biodiversity have increased, but our knowledge is still very limited. From the study carried out on literature data from the IX century to the present, it is clear that knowledge about the biodiversity of freshwater fungal communities is extremely small and fragmented in Italy. These studies mainly concern the most important lakes and rivers in northern and central Italy, while data concerning southern Italian regions and islands are almost entirely absent. Moreover, investigations are mainly focused on Ascomycota phylum, while very little information is reported about freshwater Basidiomycota. Our field observations on macrofungi along freshwater ecosystems of Italian regions showed that several species of Basidiomycota are able to grow on riparian vegetation along the banks and shores of rivers and lakes. On this regard, only a few studies in Europe focused on the presence of macromycetes in freshwater habitats. As previously mentioned, Bizio and Borsato [53] conducted a study on the mycoflora growing in the wetlands of Cansiglio Forest (Veneto, Italy) and reported the presence of 145 species of Basidiomycota. Among them, they reported *Amanita vaginata*, *Bjerkandera adusta*, *Bovista plumbea*, and *Calocybe gambosa*, also observed in our survey. Other studies were conducted in Eastern Europe about the presence of Basidiomycota in freshwater habitats of Hungary, Romania and Slovakia. According to our observations, Lazar and coworkers [59], and Chinan [60] reported the presence of several ectomycorrhizal fungi, such as *Amanita* spp. and *Lactarius* spp., growing on peat bogs of Romania. Moreover, Albert and coworkers [61] also reported the presence of *Lactarius* spp., *Leccinum* spp., and *Gymnopus dryphilus* growing in association with *Picea abies*, *Betula pubescens,* and unidentified leaves, respectively, located on different floating mats of Carpathian Basin, confirming the ability of these fungi to grow in several freshwater ecosystems.

Our field observations, moreover, highlighted that, among the Italian regions, Sicily contains the highest biodiversity among fungal species growing along its major rivers, and southern Italian regions also need to be explored to improve knowledge about this ecological group of fungi. Previous studies conducted by Ferraro and coworkers [32] have demonstrated that Sicily can be considered very rich in biodiversity regarding terrestrial fungal species and, from our observations, the same statement can be made regarding freshwater basidiomycetes. In this regard, the high number of species found on the riparian vegetation of Sicilian rivers can be explained by the favorable climatic conditions of the island.

Concerning the ecological categories to which the observed species belong, their massive presence on wood and litter as saprotrophs demonstrates their fundamental role in the decomposition of organic matter and in nutrient cycling, sustaining other freshwater organisms. In general, it is well known that, thanks to their ability to produce ligninolytic enzymes, the main role of freshwater fungi is the degradation of plant material such as leaves, wood, and stems, also in submerged conditions.

Finally, it is important to highlight that our survey on macromycetes, in addition to increasing the knowledge about this group of fungi in Italy, revealed the presence in Sicily and Campania of *Rhodotus palmatus*, a rare and endangered macromycete with very few known localities in the Italian peninsula.

## 6. Conclusions

In this study, detailed research was carried out on the state of the art of knowledge of freshwater fungi reported from the 19th century to the present in Italy. What emerged highlighted that this knowledge, at present, is very poor. Moreover, the absence of work concerning southern Italy and the islands (Sicily and Sardinia) further emphasizes that the number of species belonging to this ecological category of fungi is extremely underestimated. Very little information, moreover, has emerged regarding the phylum Basidiomycota. Our field observations aimed to reduce this knowledge gap, and showed that several species of Basidiomycota can be found, associated with riparian vegetation growing along rivers and lakes banks of Italy, and southern Italian regions, such as Sicily, are an important source of freshwater fungal biodiversity, in which it is possible to find rare and endangered fungal species (e.g., *Rhodotus palmatus*). Considering that Italy is the country with the largest presence of freshwater environments and that many of these remain unexplored, and considering the strong impact that these fungal communities have on freshwater ecosystems, more studies and research are needed to obtain more data regarding their biodiversity in Italian territories.

## Figures and Tables

**Table 1:** Binomial, substrate of isolation, and locality of freshwater macromycetes reported in the literature.

Taxa	Substrate of isolation	Locality	References
Chytridiomycetes			
*Sorokinocystis mirabilis*	Unidentified decaying wood	Canal Grande, Veneto	[46]
Dothideomycetes			
*Alternaria* sp.	Chestnut and oak decaying leaves	Alpine streams, Piemonte	[54]
*Aquanectria penicillioides*	Unidentified leaves	Bracciano Lake, Lazio	[51]
*Anguillospora crassa*	Water	Stream in Valsesia, Piemonte; Ticino Park channel, Lombardia	[49,55]
*Anguillospora longissima*	Water	Stream in Valsesia, Piemonte; Ticino Park channel, Lombardia	[49,55]
*Clavariopsis aquatica*	Water	Stream in Valsesia, Piemonte; Ticino Park channel, Lombardia	[49,55]
*Cladosporium* sp.	Chestnut and oak decaying leaves	Alpine streams, Piemonte	[54]
*Cladosporium cladosporioides*	Leaves and roots of *Phragmites australis* (Cav.) Trin. ex Steud	Trasimeno Lake, Umbria	[50]
*Davidiella tassiana*	Leaves and roots of *P*. *australis*	Trasimeno Lake, Umbria	[50]
*D. macrospora*	Leaves of *P. australis*	Trasimeno Lake, Umbria	[50]
*Dissoconium* sp.	Roots of *P. australis*	Trasimeno Lake, Umbria	[50]
*Leptosphaeria* sp.	Roots of *P. australis*	Trasimeno Lake, Umbria	[50]
*L. acuta*	*Urtica dioica* L. stem	Pian del Cansiglio, Veneto	[53]
*L. doliolum*	*Angelica sylvestris* L. stem	Pian del Cansiglio, Veneto	[53]
*Mycocentrospora aquatica*	Water	Ticino Park channel, Lombardia	[49]
*Nodulosphaeria cirsii*	Rotting stems of *Cirsium arvense* (L.) Scop. and *C*. *palustre* (L.) Scop.	Pian del Cansiglio, Veneto	[53]
*Pycnidiophora dispersa*	Leaves of *P. australis*	Trasimeno Lake, Umbria	[50]
*Ramichloridium apiculatum*	Roots of *P. australis*	Trasimeno Lake, Umbria	[50]
*Tripospermum* sp.	Water	Ticino Park channel, Lombardia	[50]
*T. myrti*	Water	Stream in Valsesia, Piemonte	[55]
*Westerdykella multispora*	Leaves of *P. australis*	Trasimeno Lake, Umbria	[50]
Eurotiomycetes			
*Aspergillus affinis*	Submerged vegetable litter (*Alnus glutinosa* L., *Salix alba* L., *Populus tremula* L., *P*. *nigra* L.)	Sacco River, Lazio	[52]
*Penicillium aculeatum*	Leaves of *P. australis*	Trasimeno Lake, Umbria	[50]
*P. atramentosum*	Roots of *P. australis*	Trasimeno Lake, Umbria	[50]
*P. brevistipitatum*	Roots of *P. australis*	Trasimeno Lake, Umbria	[50]
*P. chrysogenum*	Roots of *P. australis*	Trasimeno Lake, Umbria	[50]
*P. concentricum*	Roots of *P. australis*	Trasimeno Lake, Umbria	[50]
*P. coprophilum*	Roots of *P. australis*	Trasimeno Lake, Umbria	[50]
*P. pinophilum*	Leaves of *P. australis*	Trasimeno Lake, Umbria	[50]
*P. turbatum*	Roots of *P. australis*	Trasimeno Lake, Umbria	[50]
*Talaromyces flavus*	Leaves and roots of *P. australis*	Trasimeno Lake, Umbria	[50]
Leotiomycetes			
*Pseudagerita ossiformis*	Decaying twig of *Quercus* in a small river	Monticiano, Tuscany	[47]
*Niptera tephromelas*	*Typha angustifolia* L., *Juncus conglomeratus* L.	Collecchio Lakes, Emilia-Romagna	[56]
*Alatospora pulchella*	Unidentified leaves	Bracciano Lake, Lazio; Stream in Valsesia, Piemonte; Ticino Park channel, Lombardia	[45,49,55]
*A. acuminata*	Water	Bracciano Lake, Lazio; Stream in Valsesia, Piemonte; Ticino Park channel, Lombardia	[45,49,55]
*Articulospora grandis*	Water	Ticino Park channel, Lombardia	[49]
*A. inflata*	Water	Stream in Valsesia, Piemonte; Ticino Park channel, Lombardia	[49,55]
*A. tetracladia*	Water	Stream in Valsesia, Piemonte; Ticino Park channel, Lombardia	[49,55]
*Capitotricha bicolor*	Stem of *Senecium* sp.	Pian del Cansiglio, Veneto	[53]
*Chlorociboria aeruginascens*	Stem of *Senecium* sp.	Pian del Cansiglio, Veneto	[53]
*Crocicreas dolosellum*	*Deschampsia cespitosa* (L.) Beauv., *Carex rostrata* Stokes	Pian del Cansiglio, Veneto	[53]
*Flagellospora curvula*	Water	Stream in Valsesia, Piemonte	[55]
*Heterosphaeria patella*	Umbrelliferae stem	Pian del Cansiglio, Veneto	[53]
*Hymenoscyphus calyculus*	Rotting branch of *Alnus incana* (L.) Moench and *Picea abies Picea abies* (L.) H. Karst., 1881	Pian del Cansiglio, Veneto	[53]
*H. herbarum*	*Cirsium eriophorum* (L.) Giovanni Antonio Scopoli, 1772	Pian del Cansiglio, Veneto	[53]
*H. janthinum*	*P. abies* strobilus	Pian del Cansiglio, Veneto	[53]
*H. repandus*	Rotting stem of *Filipendula ulmaria* (L.) Maxim., 1879; branch of *Salix caprea* L.	Pian del Cansiglio, Veneto	[53]
*Lachnum clandestinum*	*Veratrum* sp. stem	Pian del Cansiglio, Veneto	[53]
*L. tenuissimum*	Rotting leaves of *C. rostrata*	Pian del Cansiglio, Veneto	[53]
*Lemonniera* sp.	Chestnut and oak decaying leaves	Alpine streams, Piemonte	[54]
*L. aquatica*	Water	Ticino Park channel, Lombardia	[49]
*L. centrosphaera*	Water	Stream in Valsesia, Piemonte	[55]
*L. filiformis*	Water	Ticino Park channel, Lombardia	[49]
*L. terrestris*	Water	Stream in Valsesia, Piemonte	[55]
*Leotia lubrica*	Litter and moss	Pian del Cansiglio, Veneto	[53]
*Lophodermium arundinaceum*	*Cirsium oleraceum* (L.) Scop. stem	Pian del Cansiglio, Veneto	[53]
*Mollisia cinerea*	*A. incana*	Pian del Cansiglio, Veneto	[53]
*M. humidicola*	*C. rostrata* leaves	Pian del Cansiglio, Veneto	[53]
*M. juncina*	*C. rostrata* rotting leaves	Pian del Cansiglio, Veneto	[53]
*M. ventosa*	*A. incana*	Pian del Cansiglio, Veneto	[53]
*Phiale* sp.	Chestnut and oak decaying leaves	Alpine streams, Piemonte	[54]
*Pyrenopeziza revincta*	*A. sylvestris*	Pian del Cansiglio, Veneto	[53]
*P. plicata*	*C. rostrata*	Pian del Cansiglio, Veneto	[53]
*Spirosphaera lignicola*	Unidentified submerged decaying twigs in stream	Monticiano, Tuscany	[48]
*Tetrachaetum elegans*	Water	Stream in Valsesia, Piemonte; Ticino Park channel, Lombardia	[49,55]
*Tetracladium* sp.	Chestnut and oak decaying leaves	Alpine streams, Piemonte	[54]
*Tetracladium marchalianum*	Water	Ticino Park channel, Lombardia	[49]
*T. setigerum*	Water	Ticino Park channel, Lombardia	[49]
*Tricladium* sp.	Water	Ticino Park channel, Lombardia	[49]
*Tricladium attenuatum*	Water	Stream in Valsesia, Piemonte	[55]
*T. splendens*	Water	Stream in Valsesia, Piemonte	[55]
*Varicosporium delicatum*	Water	Ticino Park channel, Lombardia	[49]
*V. elodeae*	Water	Stream in Valsesia, Piemonte; Ticino Park channel, Lombardia	[49,55]
Laboulbeniomycetes			
*Autoicomyces fragilis*	*Laccobium scutellaris* Olivier (Hydrophilidae)	Conegliano, Veneto	[46]
*Chitonomyces elongatus*	*Laccophilus* sp. (Dytiscidae)	De Tarzo Lake, Veneto	[46,57]
*C. ensifer*	*Laccophilus virescens* Csiki	Conegliano, Veneto	[46]
*Thripomyces italicus*	*Hydraena* sp. (Hydrophilidae)	Conegliano, Veneto	[46,58]
Pezizomycetes			
*Actinosporella megalospora*	Water	Ticino Park channel, Lombardia	[49]
*Discina accumbens*	*P. abies* litter	Pian del Cansiglio, Veneto	[53]
*D. ancilis*	*P*. *abies* basal trunk	Pian del Cansiglio, Veneto	[53]
*Scutellinia crinita*	*P*. *abies* basal trunk	Pian del Cansiglio, Veneto	[53]
*Tarzetta catinus*	*P*. *abies* basal trunk	Pian del Cansiglio, Veneto	[53]
*Campylospora chaetocladia*	Water	Ticino Park channel, Lombardia	[49]
*C. parvula*	Water	Ticino Park channel, Lombardia	[49]
*Culicidospora aquatica*	Water	Stream in Valsesia, Piemonte	[55]
*C. gravida*	Water	Stream in Valsesia, Piemonte	[55]
*Dendrospora erecta*	Water	Stream in Valsesia, Piemonte	[55]
*Heliscella stellata*	Water	Stream in Valsesia, Piemonte; Ticino Park channel, Lombardia	[49,55]
*Lunulospora curvula*	Water	Ticino Park channel, Lombardia	[49]
*Polycladium equiseti*	Water	Ticino Park channel, Lombardia	[49]
*Pyramidospora casuarinae*	Water	Ticino Park channel, Lombardia	[49]
*Speiropsis* sp.	Water	Ticino Park channel, Lombardia	[49]
*Triscelophorus monosporus*	Water	Ticino Park channel, Lombardia	[49]
*Tumularia aquatica*	Water	Ticino Park channel, Lombardia	[49]
Sordariomycetes			
*Acremonium* sp.	Leaves and roots of *P. australis*	Trasimeno Lake, Umbria Alpine streams, Piemonte	[50,54]
*Adisciso* sp.	Chestnut and oak decaying leaves	Trasimeno Lake, Umbria; Alpine streams, Piemonte	[50,54]
*Apodus oryzae*	Roots of *P. australis*	Trasimeno Lake, Umbria	[50]
*Beauveria felina*	Leaves and roots of *P. australis*	Trasimeno Lake, Umbria	[50]
*Biscogniauxia mediterranea* Kuntze	Roots of *P. australis*	Trasimeno Lake, Umbria	[50]
*Clavatospora longibrachiata*	Water	Stream in Valsesia, Piemonte; Ticino Park channel, Lombardia	[49,55]
*Diatrype disciformis*	Rotting branch of *Fagus sylvatica* L.	Pian del Cansiglio, Veneto	[53]
*Fusarium incarnatum*	Leaves and roots of *P. australis*	Trasimeno Lake, Umbria	[50]
*F. equiseti*	Leaves and roots of *P. australis*	Trasimeno Lake, Umbria	[50]
*Gibberella moniliformis*	Leaves and roots of *P. australis*	Trasimeno Lake, Umbria	[50]
*G. fujikuroi*	Leaves and roots of *P. australis*	Trasimeno Lake, Umbria	[50]
*Graphium pseudormiticum*	Leaves and roots of *P. australis*	Trasimeno Lake, Umbria	[50]
*G. penicillioides*	Leaves and roots of *P. australis*	Trasimeno Lake, Umbria	[50]
*Heliscus lugdunensis*	Water	Stream in Valsesia, Piemonte; Ticino Park channel, Lombardia	[49,55]
*Hypocrea koningii*	Leaves and roots of *P. australis*	Trasimeno Lake, Umbria	[50]
*H. lixii*	Roots of *P. australis*	Trasimeno Lake, Umbria	[50]
*Hypoxylon fuscum*	*A. incana* branches	Pian del Cansiglio, Veneto	[53]
*Lasiosphaeria hispida*	Roots of *P. australis*	Trasimeno Lake, Umbria	[50]
*Tolypocladium ophioglossoides*	Moss and *Sphagnum*	Pian del Cansiglio, Veneto	[53]
*Trichoderma aureoviride*	Roots of *P. australis*	Trasimeno Lake, Umbria	[50]
*T. harzianum*	Roots of *P. australis*	Trasimeno Lake, Umbria	[50]
*T. saturnisporum*	Roots of *P. australis*	Trasimeno Lake, Umbria	[50]
*Xylaria filiformis*	*A. sylvestris* stem	Pian del Cansiglio, Veneto	[53]
*X. hypoxylon*	*F. sylvatica* branch	Pian del Cansiglio, Veneto	[53]
*Zopfiella latipes*	Roots of *P. australis*	Trasimeno Lake, Umbria	[50]
*Z. marina*	Roots of *P. australis*	Trasimeno Lake, Umbria	[50]
*Incertae sedis*			
*Triscelophorus acuminatus*	Unidentified leaves	Bracciano Lake, Lazio	[51]
*Camposporium pellucidum*	Water	Ticino Park channel, Lombardia	[49]

**Table 2:** Species of freshwater macromycetes collected during field observations in riparian forest growing along riverbanks and banks of lakes in Italy.

Taxa	Habitat	Ecological Categories	Regions
*Amanita vaginata* (Bull.) Lam	Mixed forest: *Alnus cordata*, *Carpinus betulus*, *Fraxinus ornus*	Ectomycorrhizal	Sicily (Imera river), Liguria (Stura river), Lombardia (Lugano lake Varese, Bernigolo lake)
*Armillaria mellea* (Vahl) P. Kumm	Stumps of *Populus alba*, *Salix alba*, *Tamarix africana*, *Alnus cordata*	Necrotroph parasite	Sicily (Imera, Simeto and Alcantara rivers), Calabria (Crati river), Campania (Agnano lake, Cratere degli Astroni), Tuscany (Merse river), Piemonte (Toce river), Lombardia (Ticino river, Varese Lake), Emilia-Romagna (Po river)
*A. tabescens* (Scop.) Emel	Stumps of *Populus alba*, *Salix alba*	Necrotroph parasite	Sicily (Imera, Simeto and Alcantara rivers)
*Astraeus hygrometricus* (Pers.) Morgan	Mixed forest: *Tamarix africana*, *Salix alba*, *Populus alba*	Terricolous saprotroph	Sicily (Imera, Simeto and Alcantara rivers) Calabria (Crati river), Campania (Agnano lake, Cratere degli Astroni), Lazio (Bracciano lake), Tuscany (Isola Santa Lake), Liguria, Piemonte (Torrente Chisone, Torrente Elvo)
*Auricularia auricula-judae* (Bull.) Quél.	Stumps and trunks of *Populus alba*, *Salix alba*	Saprotroph on wood	Sicily (Imera, Belice, Salso, Oreto, Anapo, Irminio, Torto, Simeto and Alcantara rivers), Calabria (Crati river), Lazio (Bracciano Lake), Veneto (oasi Cà di Mezzo), Lombardia (Torbiere del Serbino)
*Bjerkandera adusta* (Willd.) P. Karst.	Stumps and trunks of *Populus alba*, *Salix alba*	Necrotroph parasites	Sicily (Imera, Belice, Salso, Oreto, Anapo, Irminio, Torto, Simeto and Alcantara rivers), Lombardia (Lugano lake, Varese)
*Bovista aestivalis* (Bonord.) Demoulin	Mixed forest: *Salix alba*, *Populus alba*, *Eucalyptus camaldulensis*	Saprotroph on litter	Sicily (Irminio and Alcantara rivers), Lombardia (Lugano lake Varese)
*B. nigrescens* Pers.	Mixed forest: *Salix alba*, *Populus alba*, *Eucalyptus camaldulensis*	Saprotroph on litter	Sicily (Irminio and Alcantara rivers), Lombardia (Lugano lake Varese)
*B. plumbea* Pers.	Mixed forest: *Salix* spp., *Populus alba*, *Eucalyptus camaldulensis*, *Quercus virgiliana*	Saprotroph on litter	Sicily (Irminio and Alcantara rivers), Lombardia (Oglio river)
*Calocybe gambosa* (Fr.) Donk	Mixed forest: *Salix alba*, *Populus alba*	Terricolous saprotroph	Sicily (Alcantara river), Trentino-Alto Adige (Avisio river)
*Calvatia excipuliformis* (Scop.) Perdeck	Mixed forest: *Alnus cordata*, *Pinus nigra*, *Castanea sativa*	Terricolous saprotroph	Sicily (Alcantara river)
*C. utriformis* (Bull.) Jaap	Mixed forest clearing: *Alnus cordata*., *Pinus nigra*, *Castanea sativa*	Terricolous saprotroph	Sicily (Alcantara river)
*Candolleomyces candolleanus* (Fr.) D. Wächt. & A. Melzer	Stumps of *Populus alba*, *Salix alba*, *Tamarix africana*, *Alnus cordata*	Saprotroph on wood	Sicily (Imera, Belice, Salso, Oreto, Anapo, Irminio, Torto, Simeto and Alcantara rivers), Molise (Oasi le Mortine), Lombardia (Oglio river, Riserva naturale Bosco dell’isola)
*Collybia dryophila* (Bull.) P. Kumm.	Mixed forest: *Alnus cordata*, *Pinus nigra*, *Castanea sativa*, *Salix alba*, *Populus alba*	Saprotroph on litter	Sicily (Imera, Belice Salso, Oreto, Anapo, Irminio, Torto, Simeto and Alcantara rivers)
*Collybiopsis peronata* (Bolton) R.H. Petersen	Mixed forest: *Salix alba*, *Populus alba*, *Alnus cordata*, *Fraxinus ornus*	Saprotroph on litter	Sicily (Irminio and Alcantara rivers, Trentino-Alto Adige (Noce river)
*Coprinus comatus* (O.F. Müll.) Pers.	Mixed forest: *Salix alba*, *Populus alba*, *Alnus alba*, *Fraxinus ornus*, *Castanea sativa*	Saprotroph on litter	Sicily (Imera, Belice, Salso, Oreto, Anapo, Irminio, Torto, Simeto and Alcantara rivers) Campania (Agnano lake, Cratere degli Astroni), Lazio (Bracciano lake), Emilia-Romagna (Enza river)
*C. disseminatus* (Pers.) Gray	Stumps of *Populus alba*, *Salix alba*	Saprotroph on litter	Sicily (Imera, Belice, Salso, Oreto, Anapo, Irminio, Torto, Simeto and Alcantara rivers)
*Crinipellis scabella* (Alb. & Schwein.) Kuyper	Twigs, dry and rotting grass	Saprotroph on litter	Sicily (Oreto, Anapo and Simeto rivers)
*Cyclocybe cylindracea* (DC.) Vizzini & Angelini	Stumps of *Populus alba*, *Salix alba*	Saprotroph on wood	Sicily (Imera, Belice, Salso, Oreto, Anapo, Irminio, Torto, Simeto and Alcantara rivers), Tuscany (Arno river, Albereta)
*Gymnopus dryophilus* (Bull.) Murrill	Mixed forest: *Salix alba*, *Populus alba*, *Alnus cordata*, *Fraxinus ornus*, *Castanea sativa*	Saprotroph on litter	Sicily (Imera, Belice, Salso, Oreto, Anapo, Irminio, Torto, Simeto and Alcantara rivers)
*Hohenbuehelia mastrucata* (Fr.) Singer	Clearings of *Tamarix africana*	Terricolous saprotroph	Sicily (Imera river)
*Hypsizygus ulmarius* (Bull.) Redhead	Stumps of *Populus alba*, *Salix alba*	Saprotroph on wood	Sicily (Imera, Belice, Salso, Oreto, Anapo, Irminio, Torto, Simeto and Alcantara rivers), Lombardia (Lugano lake Varese)
*Inonotus hispidus* (Bull.) P. Karst	Trunks of *Populus alba*, *Salix alba*	Saprotroph on wood	Sicily (Imera, Belice, Salso, Oreto, Anapo, Irminio, Torto, Simeto and Alcantara rivers), Trentino- Alto Adige (Passirio river)
*I. tamaricis* (Pat.) Maire	Stumps and trunks of *Tamarix africana*, *Populus alba*	Saprotroph on wood	Sicily (Imera, Belice, Salso, Oreto, Anapo, Irminio, Torto, Simeto and Alcantara rivers)
*Lactarius controversus* Pers.	Mixed forest: *Salix alba*, *Populus alba*, *Alnus cordata*, *Fraxinus ornus*, *Castanea sativa*	Ectomycorrhizal	Sicily (Imera, Torto and Alcantara rivers), Lombardia (Oglio river, Riserva naturale Bosco dell’isola), Trentino-Alto Adige (Adige river)
*Leccinum aurantiacum* (Bull.) Gray	Mixed forest: *Salix alba*, *Populus alba*, *Alnus cordata Fraxinus ornus*, *Castanea sativa*	Ectomycorrhizal	Sicily (Torto and Alcantara rivers)
*L. duriusculum* (Schulzer ex Kalchbr.) Singer	Mixed forest: *Salix alba*, *Populus alba*, *Alnus cordata Fraxinus orunus*, *Castanea sativa*	Ectomycorrhizal	Sicily (Torto and Alcantara rivers)
*Lentinus tigrinus* (Bull.) Fr.	Mixed forest: *Salix alba*, *Populus alba*, *Alnus cordata*, *Fraxinus ornus*, *Castanea sativa*	Saprotroph on wood	Sicily (Torto and Alcantara rivers), Emilia-Romagna (Po river), Lombardia (Oglio river, Varese lake park),
*Macrolepiota excoriata* (Schaeff.) Wasser	Mixed forest: *Salix alba*, *Populus alba*, *Alnus cordata*, *Fraxinus ornus*, *Castanea sativa*	Saprotroph on litter	Sicily (Imera, Belice, Salso, Oreto, Anapo, Irminio, Torto, Simeto and Alcantara, Lazio (Selva di Paliano)
*Melanoleuca polioleuca* (Fr.) Kühner & Maire	Mixed forest: *Salix alba*, *Populus alba*	Saprotroph on litter	Sicily (Imera, Salso and Oreto rivers)
*Pholiota aurivella* (Batsch) P. Kumm.	Stumps of *Populus alba*	Saprotroph on wood	Sicily (Alcantara river)
*Pleurotus cornucopiae* (Paulet) Quél.	Stumps of *Populus alba*	Saprotroph on wood	Sicily (Alcantara river), Lombardia (Lugano lake Varese)
*P. ostreatus* (Jacq.) P. Kumm	Stumps of *Populus alba*	Saprotroph on wood	Sicily (Imera, Belice, Salso, Oreto, Anapo, Irminio, Torto, Simeto and Alcantara rivers) Campania (Agnano lake, Cratere degli Astroni), Emilia-Romagna (Taro river, Po river Bosco di Porporana)
*Rhodotus palmatus* (Bull.) Maire	Woody residues of *Ulmus minor*	Saprotroph on wood	Sicily (Belice river), Campania (Agnano lake, Cratere degli Astroni)
*Schizophyllum commune* (Fr.)	Stumps and trunks of *Salix alba*, *Populus alba*	Saprotroph on wood	Sicily (Imera, Belice, Salso, Oreto, Anapo, Irminio, Torto, Simeto and Alcantara rivers)
*Schizopora paradoxa* (Schrad.) Donk	Stumps of *Populus alba*	Saprotroph on wood	Sicily (Imera, Belice, Salso, Oreto, Anapo, Irminio, Torto, Simeto and Alcantara rivers)
*Stereum hirsutum* (Willd.) Pers.	Stumps of *Populus alba*, *Salix alba*, *Tamarix africana*	Saprotroph on wood	Sicily (Imera, Belice, Salso, Oreto, Anapo, Irminio, Torto, Simeto and Alcantara rivers, Lombardia (Lugano lake Varese, Oglio river)
*Xerocomellus chrysenteron* (Bull.) Šutara	Mixed forest: *Salix alba*, *Populus alba*, *Alnus cordata*, *Fraxinus ornus*., *Castanea sativa*	Ectomycorrhizal	Sicily (Imera, Belice, Salso, Oreto, Anapo, Irminio, Torto, Simeto and Alcantara rivers), Lombardia (Lugano lake Varese)

## Data Availability

Not applicable.
